# Transient Postural Vestibulo-Cerebellar Syndrome in Three Dogs With Presumed Cerebellar Hypoplasia

**DOI:** 10.3389/fvets.2020.00453

**Published:** 2020-08-05

**Authors:** Miroslav Prikryl, Abby Caine, Viktor Palus

**Affiliations:** ^1^Jaggy Clinic, Ltd., Prague, Czechia; ^2^Neurovet, Trenčín, Slovakia; ^3^Dick White Referrals, Cambridgeshire, United Kingdom

**Keywords:** vestibular, transient, MRI, cerebellum, postural

## Abstract

This case study presents a unique transient postural vestibular syndrome in three dogs. The transient postural symptoms present as pronounced vestibulo-cerebellar signs after altering the position of the head. Magnetic resonance imaging findings of the brain suggest caudal cerebellar hypoplasia, affecting vermis, and floccular lobes bilaterally in case 1, and hypoplasia of the nodulus vermis in cases 2 and 3. No progression of clinical signs was reported in minimum of 4 months period.

## Introduction

Vestibular syndrome is a common neurological finding in dogs. It is related to a pathologic process affecting either the inner ear and/or vestibulocochlear nerve (peripherally), or the *medulla oblongata* (vestibular nuclei), brain stem, thalamus, and cerebellum (flocculonodular lobe, fastigial nuclei) centrally (1, 2). Clinically, it is characterized by signs such as head tilt, a broad based stance, nystagmus, strabismus, ataxia, and other neurological deficits related to neuroanatomical localization of the lesion. Vestibular deficits related to head movement have been described in dogs (3), introducing the relationship of nodulus and uvula pathology to vestibular signs described as positioning head tilt. The aim of our case study is to describe additional presentations related to vestibular dysfunction and cerebellar malformation.

## Case Presentation

### Case 1

**Figure 1:**
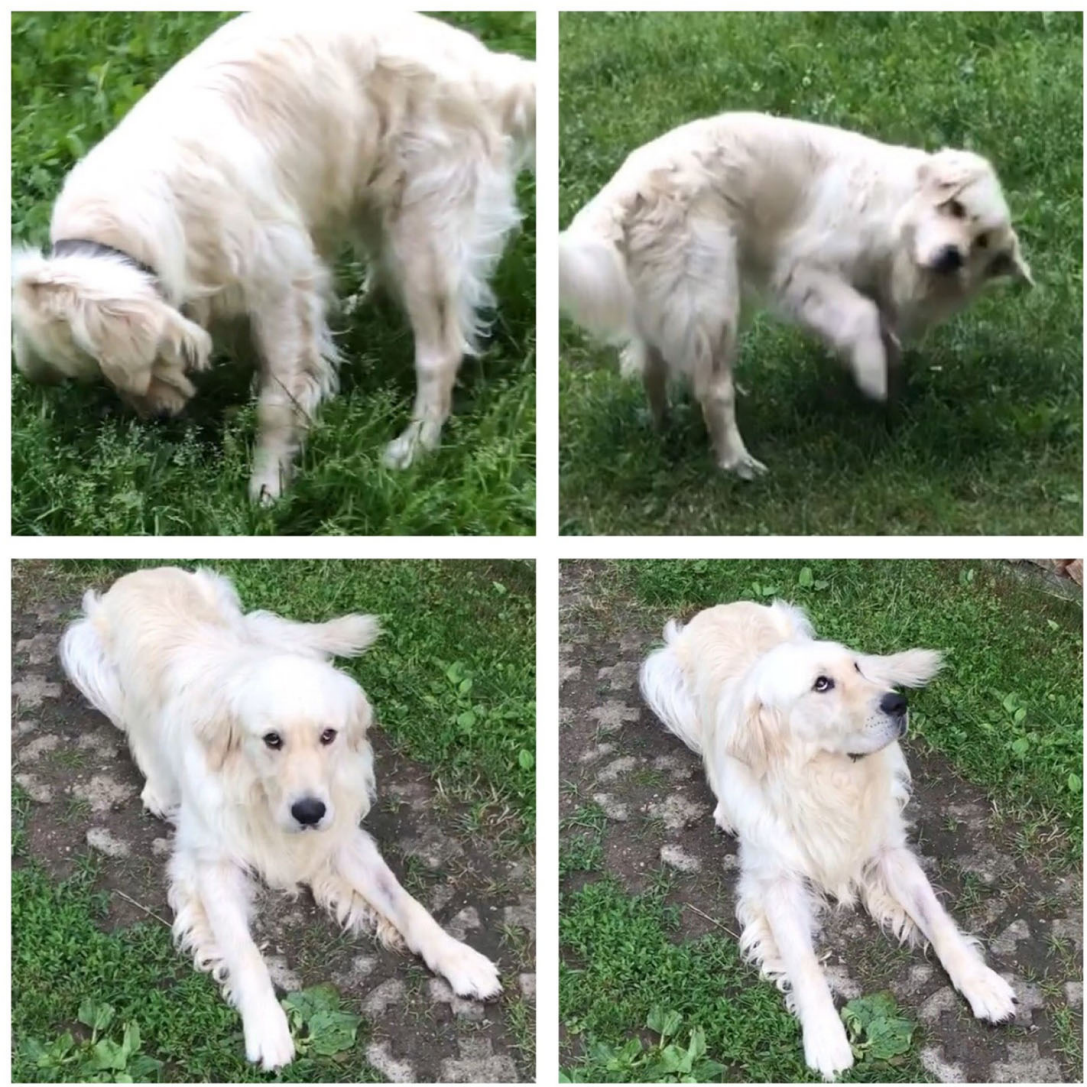
Vestibular signs of the dog in case 1. The lower images show sternal position with vertical nystagmus and inability to stand up following the extension of the neck. The upper images focusing on the sniffing behavior with abnormal body and head position (upper left) leading to transient vestibular signs (upper right).

A male Golden Retriever dog from a local shelter was presented for evaluation of incoordination and occasional head tilt. The age of the dog was estimated to be 2–3 years based on general appearance and teeth condition. No information was available regarding onset and duration of clinical signs. A general clinical examination did not reveal any abnormalities. Neurological findings included an episodic bilateral head tilt related to the posture of the head during sniffing and very mild cerebellar ataxia (hyperflexion of the limbs, mild pelvic limb incoordination and mild truncal swaying) lasting several seconds (Figure 1). Postural testing by extension of the neck revealed an exacerbation of bilateral vestibular signs with vertical nystagmus, a sternal body position and an inability to stand without signs of head tilt, lasting 10–15 s, followed by mild cerebellar ataxia lasting several seconds (Video 1). In between the episodes, the dog presented only subtle pelvic limb dysmetria. The neuroanatomic localization was consistent with a lesion affecting the vestibular system within the cerebellum. Due to limited history information, most likely differential diagnoses at the time of presentation consisted of inflammatory, degenerative, and congenital disorders, however, vascular, neoplastic, and infectious conditions were also included.

Complete blood count (ProCyte Dx Hematology analyzer), chemistry profile (Cobas c 111 analyzer), serum thyroxin level (IDEXX Catalyst), and CRP (C-reactive protein) were unremarkable. Head, thoracic, abdominal radiographs as well as abdominal and cardiac ultrasound examination were performed with no abnormalities detected, and blood pressure measurements were normal. Holter ECG (Mortara H3+) monitoring (24 h) during the episode did not reveal any abnormalities. Serological testing for neosporosis (IFAT) was within normal limits. Serological testing for toxoplasmosis revealed elevated IgG-titer 1:1,024 (normal <1:32). Magnetic resonance imaging (MRI) of the brain was scheduled in a month after admission. Interim supportive treatment with vitamins B1 (1.2 mg/kg SID), B6 (4 mg/kg SID), and B12 (0.250 mg pro toto SID) was initiated and the neurological status remained stable.

**Figure 2:**
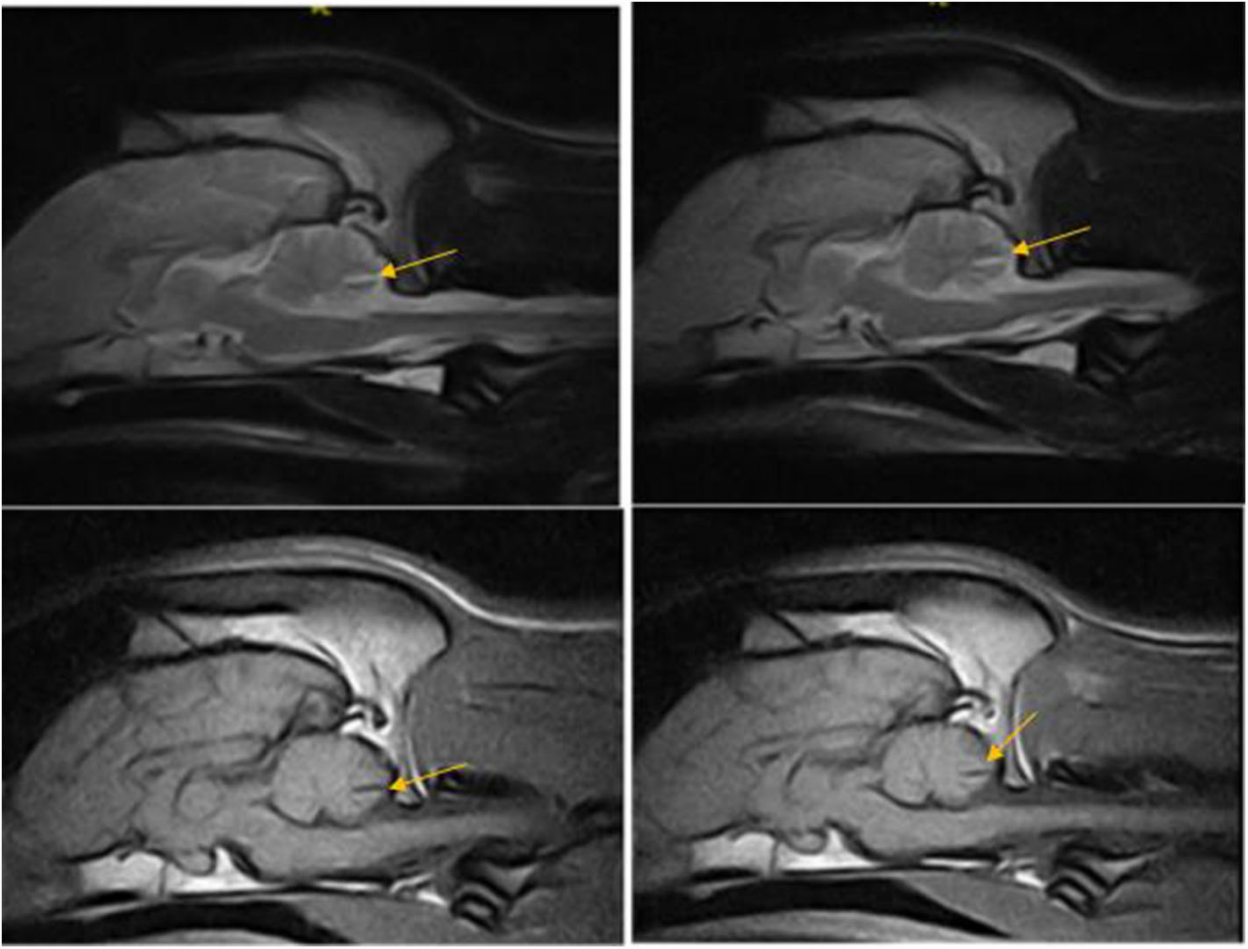
T2W and T1W sagittal images of the dog in case 1 obtained 12 months apart showing non-progressive reduction in the tissue volume in the caudal cerebellar vermis indicated by deep wide sulci between the folia, particularly noted between pyramis and tuber—(arrow).

**Figure 3:**
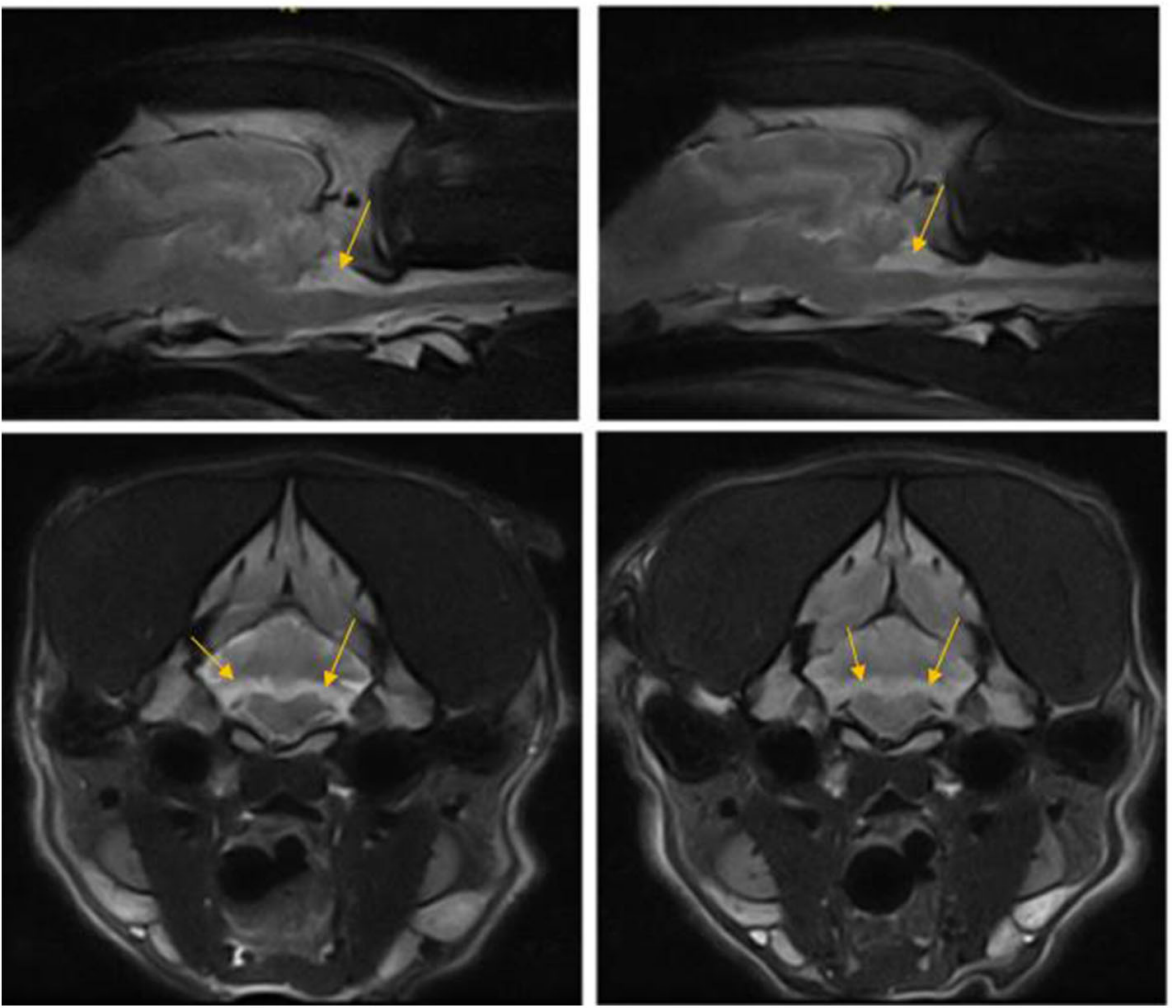
T2W parasagittal and transverse images of the dog in case 1 at the level of nodulus vermis 12 months apart showing non-progressive loss of cerebellar hemisphere tissue in the area of flocculus (arrows).

An MRI was performed under general anesthesia using a 0.18 T Esaote Vet-MR unit, used for all cases. Multiplanar T2-weighted (T2W), T1-weighted (T1W), T2W-fluid attenuated inversion recovery (T2W-FLAIR) images of the brain and cranial cervical region were acquired. Additional T1W images were acquired following intravenous administration of a gadolinium-based contrast medium (Omniscan, GE Healthcare AS, NO, 0.1 mmol/kg). The sagittal and transverse images revealed evidence of a wider and subjectively deeper sulci between the *pyramis vermis* and the *tuber vermis* consistent with caudal cerebellar hypoplasia (Figures 2, 3). Additionally, T2W parasagittal images and T2W transverse images at the level of the *nodulus vermis* showed smaller floccular lobes giving the appearance of widening of the lateral apertures. The cerebrospinal fluid (CSF) analysis [cisternal tap, TNCC, and cytology (Statspin Cytofuge 12)] was within normal limits. At this time, second serological test for *Toxoplasma gondii* was performed with IgG-titer 1:256, which remained above the limit, however had decreased. At this time, the most likely differential diagnoses were cerebellar hypoplasia due to perinatal viral infection or cerebellar abiotrophy.

After 12 months, without a change in neurological status, a repeated MRI examination of the brain was performed including short tau inversion recovery (STIR) sequences. A comparison of the previous and new MRI studies indicated no change (Figure 2). Another CSF analysis was performed (cisternal tap) including infectious disease PCR assays (*Ehrlichia canis*, canine herpes virus, canine parvovirus, canine distemper virus, *Neospora caninum, Toxoplasma gondii, and Anaplasma phagocytophilum*) and tick encephalitis antibody testing, and was within normal limits. Twenty two months since the first presentation, the dog has been stable with the transient vestibular episodes still present, mainly during playing. Cerebellar hypoplasia was considered to be the presumed diagnosis.

### Case 2

A 2-month-old female Pug was presented with a history of the vestibular episodes especially during or after eating. The owners first noticed the episodes when the puppy started to eat dry food. General physical examination was unremarkable. Neurological examination showed very mild vestibular ataxia while the puppy was walking. However, after the postural head testing, playing and eating, the vestibular deficits became transiently pronounced, showing moderate head tilt to the left, moderate vestibular ataxia with drifting to the left and a broad-based stance. After several seconds to a minute, the deficits mostly disappeared. Neuroanatomic localization was consistent with vestbulo-cerebellum. Differential diagnoses included congenital and degenerative causes, nevertheless, inflammatory and infectious conditions were considered as well. The complete blood count (IDEXX Lasercyte) and serum biochemistry results (IDEXX Catalyst, Clip 10) were normal for a puppy.

**Figure 4:**
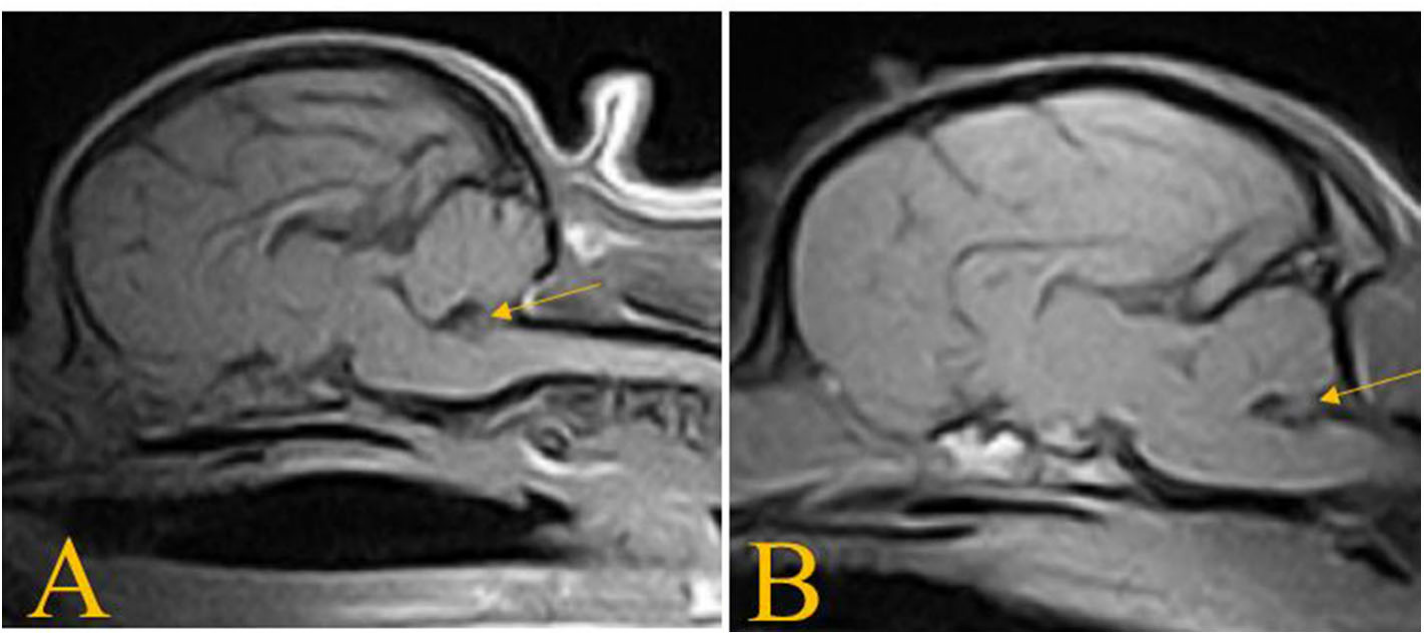
T1W sagittal images of the dogs in case 2 **(A)** and case 3 **(B)** showing absence of the nodulus vermis.

Brain MRI was performed on the initial presentation day, and multiplanar T2W, T2W-FLAIR, T1W, and T1W post intravenous administration of a gadolinium based contrast agent (Omniscan, GE Healthcare AS, NO, 0.1 mmol/kg) images were acquired. The T1W and T2W sagittal images showed a reduced size of the *nodulus* and *uvula* of the caudal cerebellum with hypoplasia of the caudal cranial fossa (Figure 4A), without contrast enhancement. The patient also had a midline fusion defect of the dorsal arch of C1, however, did not demonstrate any associated cervical instability. The cerebrospinal fluid (CSF) analysis [cisternal tap, TNCC and cytology (Statspin Cytofuge 12)] was within normal limits. Both infectious disease PCR assays (*Ehrlichia canis*, canine herpes virus, canine parvovirus, canine distemper virus, *Neospora caninum, Toxoplasma gondii*, and *Anaplasma phagocytophilum*) and tick encephalitis antibody testing from CSF were negative. Caudal cerebellar hypoplasia was suspected and no treatment was administered.

The owners report a stable status after 4 months, and the puppy has learnt to compensate for the deficits. The episodes continue to occur; mostly after playing with sticks and toys and now are rarely noted during eating.

### Case 3

A 7-month-old female Boston terrier was presented for vestibular episodes. The owners first noticed the episodes when she was 3 months of age. According to the owners, the episodes were present mostly during, or shortly after, sniffing and eating. General physical examination was unremarkable. Neurological examination showed only mild head tilt to the left during walking and standing, however, oculo-vestibular reflex testing caused marked vestibular ataxia, a broad based stance and moderate to severe head tilt to the right. This transient postural vestibular episode lasted for approximately a minute following which the dog returned to its inter-episodic status. Neuroanatomical localization was consistent with pathology related to vestibulo-cerebellum. Differential diagnoses were considered in the same manner as in case 2. The owners declined hematological and biochemical analysis.

An MRI examination of the brain was performed and T2W, T2W-FLAIR, T1W, and T1W post contrast (Omniscan, GE Healthcare AS, NO) images were acquired. The T1W and T2W sagittal images showed reduced size of the *nodulus* and *uvula* of the caudal cerebellum (Figure 4B). There was no other abnormality observed in the brain, and no abnormal contrast enhancement. Complete analysis of the cerebrospinal fluid was declined. These imaging findings were considered most likely to represent caudal cerebellar hypoplasia, and no treatment was recommended.

According to the owners, the dog is stable after 7 months, and has adapted to the episodes which remain present during playing or sniffing, lasting ~20–30 s. In between the episodes, the dog's gait and head posture is normal.

## Discussion

The cases described here presented with a unique change in severity of the vestibulo-cerebellar signs after the alteration of the head position, while having none to mild vestibulo-cerebellar signs between the episodes. Our investigation did not lead to a specific diagnosis and histopathologic confirmation of the MR findings was not available in these cases since none were euthanized. The MRI studies suggested unspecified reduction of size of the caudal cerebellar structures (caudal vermis, floccular lobes). Cases 2 and 3 share common MRI findings with absent *nodulus* as the main finding, whereas smaller floccular lobes and deeper sulci between *pyramis* and *tuber vermis* are the main findings in case 1. Clinically, the pronounced vestibular deficits in cases 2 and 3 were lateralized, while case 1 seemed to be bilateral. We were not able to prove the cause in any of the described cases, however, caudal cerebellar hypoplasia was suspected based on MRI examination.

Benign paroxysmal positional vertigo (BPPV) and central paroxysmal positional vertigo (CPPV) are well-known conditions in human medicine. While BPPV is the most common positional vestibular disturbance without an underlying cause, CPPV is related to cerebellar pathology (4). Transient vestibular signs in patients with CPPV such as vertigo and nystagmus are caused by changing the posture of the head. The underlying mechanism is not yet fully understood, however, interruption of the vestibular nuclei-archicerebellar loop seems to be responsible for central paroxysmal positional vertigo (5).

Positioning head tilt has been described in three dogs with presumptive *nodulus* and ventral *uvula* hypoplasia (3). In these dogs, the head tilted to the opposite side when the dog turned during walking, while the head was held in a level position when static or when the dog was walking in a straight line. All dogs showed absence of *nodulus* and ventral *uvula* on MRI imaging. Cases in reported study had consistent postural vestibular signs while head turning (positioning head tilt), changing depending on side turning to, however, dogs did not present additional or worsening of vestibular signs while stressing vestibular system by neck and head extension. Cases in our study are displaying very similar findings on MRI examination with some overlap in clinical presentation, nevertheless, the positional aspect is different. The patients are showing limited amount of vestibulo-cerebellar deficits during gait but there is marked to severe temporary deterioration of vestibular signs elicited by changing the head posture (positional sign). Additionally, MRI findings and vestibulo-cerebellar signs in the case 1 of our study differs even more. Mild transient vestibular deficits during normal behavior turn to severe temporary vestibulo-cerebellar signs after marked postural changes of the head. Imaging findings are also suggestive of floccular lobes atrophy bilaterally in addition to caudal vermis malformation. Therefore, despite similar MRI findings between the studies and concurrent positioning tilting of the head in case 1, neurological manifestation of posture-related vestibular signs are different with the emphasis on marked temporary deterioration caused by positional changes of the head.

Our hypothesis is related to the activity of the vestibular nuclei being continually under cerebellar control. This control includes regulation of ongoing reflexes, vestibuloocular and vestibulospinal, and an apparent role in reflex plasticity. It is the vestibulocerebellum, consisting of the flocculus, nodulus, uvula, and ventral paraflocculus, that is related particularly closely to the vestibular system and to some of the reflexes that result when the labyrinth is activated (6). The flocculonodular lobe and adjacent portions of vermal lobule IX (the paraflocculus) receive afferents from the ipsilateral vestibular ganglion (primary vestibulocerebellar fibers) and vestibular nuclei (secondary vestibulocerebellar fibers). Along with the fastigial nucleus, they form the vestibulocerebellar module. Vestibulocerebellar fibers access the flocculonodular cortex and fastigial nucleus via the juxtarestiform body and convey information concerning the position of the head and body in space as well as information useful in orienting the eyes during movements. The unipolar brush cell is largely unique to the granular layer of the vestibulocerebellum and is involved in the cerebellar and vestibular regulation of eye movement. This information is supplemented by inputs carried on olivocerebellar fibers from the contralateral olivary nuclei, and on pontocerebellar fibers (only to the flocculus) from the contralateral basilar pons. These pathways convey indirect inputs from nuclei of the diencephalon and brainstem, which are concerned with a broad spectrum of information regarding visual processing and eye movements (7). We believe that various pathological cerebellar conditions affecting these structures will result in disinhibition of vestibular nuclei leading to static mild vestibulo-cerebellar signs. These can be subtle and exacerbated by stressing the vestibular system by changing the head posture, leading to severe temporal deterioration of vestibular signs, which appears to be related to the pathology of vestibulocerebellum as presented in our study.

MRI findings in our case study are consistent with cerebellar tissue reduction, which can be a result of active pathologic process such as degeneration or non-progressive finding due to atrophy or hypoplasia. Cerebellar cortical abiotrophy is a degenerative, slowly progressive disorder with a genetic component described in multiple breeds (8–13). However, the repeated MRI study in case 1 and follow up in all three cases did not suggest and signs of progression as would be expected with degeneration. Cerebellar hypoplasia has been described in patients suffering from congenital diseases, such as Dandy Walker like malformation (14–18) or *in utero* viral infections (19, 20). The MRI findings of cerebellar vermis hypoplasia and cystic malformation of the 4th ventricle (termed a variant Dandy Walker malformation) described in dogs affects a greater area of the cerebellar vermis (21) than in the cases presented in this report. On the other hand, MRI findings in one of the dogs affected by *in utero* infection showed a mild increase in CSF between dorsal cerebellar folia compared to the ventral cerebellar folia on sagittal images, suggestive of folial atrophy and cerebellar hypoplasia (20). In these cases, viral infection was not identified with PCR testing of cerebrospinal fluid, however, viral DNA could possibly be amplified from brain tissue *post mortem*. Therefore, we believe that the cases presented in this current report suffer from a partial cerebellar vermis hypoplasia, rather than Dandy Walker malformation.

Vestibulo-cerebellar signs in the cases described above are considered most likely related to the imaging findings of caudal cerebellar hypoplasia, particularly affecting caudal vermis and floccular lobes, however, this cannot be proven without post mortem examination and histology. Specific treatment was not pursued in these cases, nonetheless avoidance of activities like swimming were recommended. The disorder described was non-progressive for at least the duration of the follow up period, and does not alter the overall quality of life. The main limitation of our case study is a lack of histopathological examination providing a definitive diagnosis. We believe that vestibulocerebellum plays a crucial part in regulating the vestibular system in dynamic postural changes and its dysfunction also presents with posture-related vestibular signs.

## Data Availability Statement

The raw data supporting the conclusions of this article will be made available by the authors, without undue reservation, to any qualified researcher.

## Ethics Statement

Ethical review and approval was not required for the animal study because we are presenting case study on three dogs, which underwent diagnostic testing (blood analysis, MRI of the brain, CSF tap) approved by their owners and we did not perform any experimental treatment on these animals. Written informed consent was obtained from the owners for the participation of their animals in this study.

## Author Contributions

MP has contributed to writing the article and managing the patient in Case 1. VP has contributed to expertise and knowledge and providing diagnostic options. AC has contributed to expertise in describing MRI studies in the article. All authors contributed to the article and approved the submitted version.

## Conflict of Interest

The authors declare that the research was conducted in the absence of any commercial or financial relationships that could be construed as a potential conflict of interest.
