# Corrigendum: Transient Postural Vestibulo-Cerebellar Syndrome in Three Dogs With Presumed Cerebellar Hypoplasia

**DOI:** 10.3389/fvets.2021.691042

**Published:** 2021-09-21

**Authors:** Miroslav Prikryl, Abby Caine, Viktor Palus

**Affiliations:** ^1^Jaggy Clinic, Ltd., Prague, Czechia; ^2^Neurovet, Trenčín, Slovakia; ^3^Dick White Referrals, Cambridgeshire, United Kingdom

**Keywords:** vestibular, transient, MRI, cerebellum, postural

In the original article, there was an error in the Introduction section, incorrect terminology was used. “Head posture” has been replaced by “head movement” and “postural changes of the head” has been replaced by “positioning head tilt.” The corrected paragraph appears below.

Vestibular syndrome is a common neurological finding in dogs. It is related to a pathologic process affecting either the inner ear and/or vestibulocochlear nerve (peripherally), or the *medulla oblongata* (vestibular nuclei), brain stem, thalamus, and cerebellum (flocculonodular lobe, fastigial nuclei) centrally (1, 2). Clinically, it is characterized by signs such as head tilt, a broad based stance, nystagmus, strabismus, ataxia, and other neurological deficits related to neuroanatomical localization of the lesion. Vestibular deficits related to head movement have been described in dogs (3), introducing the relationship of nodulus and uvula pathology to vestibular signs described as positioning head tilt. The aim of our case study is to describe additional presentations related to vestibular dysfunction and cerebellar malformation.

The authors have updated the Discussion section, Paragraph 3, as the original shows below.

Positional head tilt has been described in three dogs with presumptive *nodulus* and ventral *uvula* hypoplasia (3). In these dogs, the head tilted to the opposite side when the dog turned during walking, while the head was held in a level position when static or when the dog was walking in a straight line. All dogs showed absence of *nodulus* and ventral *uvula* on MRI imaging. We did not observe head tilting related to head turning while walking. Cases in reported study had consistent postural vestibular signs while head turning, changing depending on side turning to, however, the dog did not present additional or worsening of vestibular signs while stressing vestibular system by neck extension. Cases 2 and 3 in our study are displaying very similar findings on MRI examination, nevertheless, the presentation is different. The patients are showing limited amount of vestibulo-cerebellar deficits during normal gait without tilting the head to the side opposite the side they are turning to, but there is marked to severe temporary deterioration of vestibular signs elicited by changing the head posture. Additionally, MRI findings and vestibulo-cerebellar signs in the case 1 of our study differs even more. Mild transient vestibular deficits during normal behavior turn to severe temporary vestibulo-cerebellar deficits after marked postural changes of the head. Imaging findings are also suggestive of floccular lobes atrophy bilaterally in addition to caudal vermis malformation. Therefore, despite similar MRI findings between the studies, neurological manifestation of posture-related vestibular signs are different with the emphasis on marked temporary deterioration caused by head posturing.

The corrected paragraph appears below.

Positioning head tilt has been described in three dogs with presumptive *nodulus* and ventral *uvula* hypoplasia (3). In these dogs, the head tilted to the opposite side when the dog turned during walking, while the head was held in a level position when static or when the dog was walking in a straight line. All dogs showed absence of *nodulus* and ventral *uvula* on MRI imaging. Cases in reported study had consistent postural vestibular signs while head turning (positioning head tilt), changing depending on side turning to, however, dogs did not present additional or worsening of vestibular signs while stressing vestibular system by neck and head extension. Cases in our study are displaying very similar findings on MRI examination with some overlap in clinical presentation, nevertheless, the positional aspect is different. The patients are showing limited amount of vestibulo-cerebellar deficits during gait but there is marked to severe temporary deterioration of vestibular signs elicited by changing the head posture (positional sign). Additionally, MRI findings and vestibulo-cerebellar signs in the case 1 of our study differs even more. Mild transient vestibular deficits during normal behavior turn to severe temporary vestibulo-cerebellar signs after marked postural changes of the head. Imaging findings are also suggestive of floccular lobes atrophy bilaterally in addition to caudal vermis malformation. Therefore, despite similar MRI findings between the studies and concurrent positioning tilting of the head in case 1, neurological manifestation of posture-related vestibular signs are different with the emphasis on marked temporary deterioration caused by positional changes of the head.

The authors apologize for this error and state that this does not change the scientific conclusions of the article in any way. The original article has been updated.

## Publisher's Note

All claims expressed in this article are solely those of the authors and do not necessarily represent those of their affiliated organizations, or those of the publisher, the editors and the reviewers. Any product that may be evaluated in this article, or claim that may be made by its manufacturer, is not guaranteed or endorsed by the publisher.
